# Astrogliosis is delayed in type 1 interleukin-1 receptor-null mice following a penetrating brain injury

**DOI:** 10.1186/1742-2094-3-15

**Published:** 2006-06-30

**Authors:** Hsiao-Wen Lin, Anirban Basu, Charles Druckman, Michael Cicchese, J Kyle Krady, Steven W Levison

**Affiliations:** 1 Department of Neurology and Neuroscience, UMDNJ-New Jersey Medical School, Newark, NJ 07103, USA; 2 National Brain Research Centre, Gurgaon – 122 050, India; 3 Dept. of Neural and Behavioral Sciences, The Pennsylvania State University College of Medicine, Hershey, PA 17033, USA

## Abstract

The cytokines IL-1α and IL-1β are induced rapidly after insults to the CNS, and their subsequent signaling through the type 1 IL-1 receptor (IL-1R1) has been regarded as essential for a normal astroglial and microglial/macrophage response. To determine whether abrogating signaling through the IL-1R1 will alter the cardinal astrocytic responses to injury, we analyzed molecules characteristic of activated astrocytes in response to a penetrating stab wound in wild type mice and mice with a targeted deletion of IL-1R1. Here we show that after a stab wound injury, glial fibrillary acidic protein (GFAP) induction on a per cell basis is delayed in the IL-1R1-null mice compared to wild type counterparts. However, the induction of chondroitin sulfate proteoglycans, tenascin, S-100B as well as glutamate transporter proteins, GLAST and GLT-1, and glutamine synthetase are independent of IL-1RI signaling. Cumulatively, our studies on gliosis in the IL-1R1-null mice indicate that abrogating IL-1R1 signaling delays some responses of astroglial activation; however, many of the important neuroprotective adaptations of astrocytes to brain trauma are preserved. These data recommend the continued development of therapeutics to abrogate IL-1R1 signaling to treat traumatic brain injuries. However, astroglial scar related proteins were induced irrespective of blocking IL-1R1 signaling and thus, other therapeutic strategies will be required to inhibit glial scarring.

## Background

The cytokines interleukin-1α and interleukin-1β (collectively referred to as IL-1) are dramatically and rapidly induced following injury to the CNS and elevated IL-1 levels are associated with many neurodegenerative diseases [1]. For instance, IL-1β is rapidly induced in experimental models of stroke [2,3] and mice that have decreased IL-1 production are significantly protected from ischemic injury [4-7]. Similarly, administering IL-1 receptor antagonist or IL-1β blocking antibodies reduces neuronal death subsequent to ischemia [8-10]. There also is increased IL-1β production surrounding amyloid plaques in brains of patients with Alzheimer's disease and Down Syndrome [11], and IL-1 has been implicated in the excessive production and processing of beta-amyloid precursor protein as well as the synthesis of most of the known plaque-associated proteins [12]. IL-1 also has been shown to be elevated in the spinal fluid and within demyelinated lesions of patients with multiple sclerosis (MS) [13-15].

Microglia appear to be the earliest and major source of IL-1 after CNS injury, infection or inflammation, and they express caspase-1, the enzyme responsible for converting pro-IL-1β to its active form [16]. IL-1 subsequently increases the production of inflammatory mediators, such as cyclooxygenase 2, prostanoids, nitric oxide, matrix metalloproteinases, collagenase [17], and pro-inflammatory cytokines, including Interleukin-6 (IL-6) [18,19], tumor necrosis factor alpha (TNF-α) [20], colony stimulating factors [21] as well as itself. These molecules subsequently establish a feedforward cycle of inflammation [6].

Contrary to accumulating evidence that portrays IL-1 as a maladaptive injury related cytokine IL-1 increases the expression of multiple growth and trophic factors, including fibroblast growth factor-2 [22], transforming growth factor β 1 [23], ciliary neurotrophic factor [24], nerve growth factor (NGF) [25-28], insulin-like growth factor-1 [29] and hepatocyte growth factor [30], and these factors can promote the survival of neurons and glia.

Determining which cellular and molecular responses to CNS injury are coordinated by IL-1 signaling is essential towards a better understanding of how antagonizing IL-1 protects neurons from injury and disease. In several studies we showed that IL-1 signaling through the type 1 IL-1 receptor (IL-1R1) is essential for multiple aspects of the brain's response to a tissue damaging injury. Analyses at both cellular and molecular levels to a penetrating neocortical injury in mice that lack IL-1R1 demonstrated: diminished responsiveness of macrophages and microglia, deficient recruitment of peripheral macrophages, attenuated production of the vascular cell adhesion molecule-1 (VCAM-1), attenuated cyclooxygenase-2 production and attenuated levels of pro-inflammatory cytokine mRNAs. By contrast, the induction of NGF was intact [31]. Furthermore, studies on IL-1R1-null mice following a mild stroke demonstrated that abrogating IL-1R1 signaling reduces edema, recruitment of immune cells, production of several proinflammatory cytokines as well as microglial activation and therefore leads to reduced brain damage and preserved neurological functions [32,33]. In another study we demonstrated that the expression of ceruloplasmin (CP) is induced by a traumatic injury and that IL-1 is responsible for the injury-induced expression of CP in astrocytes [34].

To investigate whether IL-1 signaling through IL-1R1 abrogates the fundamental responses of astrocytes to a penetrating injury, here we have analyzed a panel of molecules associated with astrocytic functions. We analyzed the expression of the structural protein GFAP as increases in this protein support the integrity of the parenchyma after damage and GFAP-null mice are more susceptible to injuries than their wild type counterparts [35,36]. We also analyzed levels of glutamate transporters and the glutamate catabolic enzyme glutamine synthetase, since the capacity of an astrocyte to remove glutamate from the extracellular space will affect amino acid induced excitotoxicity [37]. As astrocytes also buffer levels of brain calcium and as the calcium binding protein S-100B also has neurotrophic properties [38-40], we measured the levels of S-100B after injury. We also examined the expression of the protease-activated receptor 1 (PAR-1) in wild type (WT) and IL-1R1-null mice following a neocortical penetrating injury as this receptor has been implicated in astrocyte hyperplasia after brain injury [41]. Last, we analyzed the expression of several extracellular matrix proteins that are known constituents of the astroglial scar to assess whether scar formation will be reduced in the absence of IL-1R1 signaling.

## Methods

### Experimental animals

Adult male IL-1R1-null mice backcrossed 9 times against a C57BL/6 background and C57BL/6 WT mice were used between 3 and 12 months of age. IL-1R1-null mice were originally provided by Amgen Inc (Seattle, WA). All mice were bred and maintained at the Hershey Medical Center by the Department of Comparative Medicine, an AAALAC accredited facility. Animal experimentation was in accordance with research guidelines set forth by Penn State University and the Society for Neuroscience Policy on the Use of Animals in Neuroscience Research.

### Penetrating brain injury and micro-injection of IL-1

Surgery on adult male mice was performed under xylazine/ketamine anesthesia (2mg xylazine and 15 mg ketamine/kg). Once the animal failed to respond to an external stimulus such as a toe pinch, it was secured in a stereotactic apparatus. A midline incision exposed the skull and a small hole of 1.35 mm in diameter was drilled through the skull. Three 1 mm deep penetrating stab wounds were produced perpendicular to the pial surface with a 45° angle 26-gauge needle. The lesion site remained constant at 2.0 mm caudal and 2.0 mm lateral from Bregma. Overall the procedure took 30 minutes per animal. The burr hole was filled with gel-foam and the scalp was sutured. The animals were placed on a warming mat, allowed to recover, and then returned to the animal facility. At intervals, the mice were sacrificed by cervical dislocation. To insure reproducible diameter tissue sampling, the area of the cortex containing the stab wound and adjacent tissue was removed using a 2.7 mm trephine. In addition, tissue from the same location relative to Bregma in the opposite hemisphere was removed and used as a control. From this sample any subcortical structures were removed, isolating only the neocortex and adjacent white matter. The samples were placed in plastic tubes, quick-frozen on dry ice and stored at -80°C until assayed.

For the micro-injection procedure a sterile glass micro-pipette (diameter < 50 μm) was used to inject 5 units (in a volume of 2 μl) of recombinant murine IL-1β (R&D Systems, Inc, Minneapolis) into the cortex. The area of surgery and the other measures following the surgery are identical for both stab wound injury and micro-injection.

### Immunohistochemistry and histological analysis

Animals used for immunocytochemistry for GFAP staining were perfused with culture medium containing 7 U/ml heparin followed by a fixative containing 3% paraformaldehyde and 0.1% glutaraldehyde in phosphate buffer, pH 7.35. Brains were dehydrated through graded alcohols and embedded in paraffin wax. Sections were cut at 6 μm and mounted onto Superfrost+ slides. Prior to staining, sections were de-waxed using standard methods and Immunocytochemistry was performed as described previously [42]. Counts of GFAP+ cells were performed on photomicrographs taken at 40 × magnification in regions 240 μm away from the lesion site of brain sections from WT (n = 4) and IL-1R1-null (n = 3) animals at day 3. Four to five pictures per section were taken. The number of GFAP+ astrocytes from each picture was counted by an investigator blinded to their identity.

### Cell culture

Primary astrocyte and microglial cultures were prepared from newborn C57BL/6 mice (P0-2). Pups were sacrificed by decapitation and the whole brain excluding the cerebellum was isolated. The meninges were removed, the tissue was enzymatically and mechanically dissociated and the cell suspension was passed through 100 μm and 40 μm nylon mesh screens sequentially. Cells were counted using a hemocytometer in the presence of 0.1% trypan blue. Mixed glial cultures were plated into 75 cm^2 ^tissue culture flasks at a density of 1 × 10^5 ^viable cells/cm^2^. Cells were fed with MEM-C (10% fetal bovine serum (FBS), 2 mM glutamine, 100U/100 μg/ml penicillin and streptomycin and 0.6% glucose in Eagles minimum essential media). The medium was changed every two days after plating.

To establish enriched astrocytes, the original flasks were shaken overnight to remove contaminating O-2A progenitors and microglia. The adherent astrocytes and the mixed glia from original flasks were replated into 6 well plates at a density of 3 × 10^4 ^viable cells/cm^2 ^fed with MEM-C. After reaching confluence, the cells were maintained in a chemically defined medium (MN1A) (Dulbecco's modified eagle's medium/F12 with 15 mM HEPES and 1 mm L-glutamine, 5 ng/ml insulin, 20 nM progesterone, 100 μM putrescine, 5 ng/ml selenium, 50 U/50 ng/ml Penicillin/Streptomycin, and 50 μg/ml apo-transferrin) for four days. To establish enriched primary cultures of cortical neurons, the cortices from brains of 17-day-old mouse embryos were dissociated by trituration, layered onto a 4% BSA gradient and centrifuged at 700 × g for 2 min. The cells were resuspended in L-15 medium containing supplements [43] and plated on poly-l-ornithine coated dishes at a density of 6 × 10^4 ^cells/cm^2 ^in 2 ml on 60 mm petri dishes. One day after plating, media were replaced with neurobasal medium supplemented with B-27, 6.3 mg/ml NaCl, and 10 U/ml penicillin/streptomycin. The cells were maintained in vitro for 10 days to allow the neurons to differentiate. The purity of the cultures was assessed by determining the percentage of GFAP (1:500, DAKO, Carpinteria, CA) immunoreactive cells (<5%). Media and B-27 were purchased from Gibco (Rockville, MD). Other chemicals were obtained from Sigma (St. Louis, MO).

Astrocytes, mixed glia and cortical neurons were treated with 5 ng/ml of recombinant murine IL-1β (rmIL-1β) (R & D Systems, Minneapolis, MN) in defined medium for 24 hrs, then washed twice with ice-cold PBS, and lysed in buffer containing a final concentration of 1% Triton-X 100, 10 mM Tris-HCl, pH 8.0, 150 mM NaCl, 0.5% nonidet P-40, 1 mM EDTA, 0.2% EGTA, 0.2% sodium orthovanadate and protease inhibitor cocktail (Sigma, St Louis, MO). The lysate was gently agitated for 15 minutes at 4°C. DNA was sheared using a 21-gauge needle and then the homogenate was centrifuged at 10,000 rpm for 15 minutes at 4°C. Protein levels were determined using the BCA colorimetric assay (Pierce, Rockford, IL). Protein lysates were aliquoted and stored at -80°C until needed. Control cells received defined medium, minus cytokine.

### ELISA

Stab wounds were performed on adult WT C57BL/6 and IL-1R1 knockout (KO) mice as described above. Mice were sacrificed at 3, 5, 7 and 10 days following injury. Cortical tissues were placed in 1.5 ml microcentrifuge tubes with 150 μl of homogenization buffer (20 mM Tris, 1 mM EDTA, 255 mM sucrose with protease inhibitor cocktail (aprotinin, leupeptin, pepstatin and AEBSF) from Sigma (1 ml of cocktail per 20 g cells wet weight). Samples were homogenized and then sonicated for 10 pulses 2× each. Protein concentrations were determined using the Pierce BCA Protein Assay Kit. All tissue samples were stored at -80°C until needed. ELISA for GFAP was performed using a two-site ELISA as described previously [44].

### Western blotting

For immunoblotting of chondroitin sulphate proteoglycan-4 (CSPG-4), 2.5 μg of protein was digested with chondroitinase ABC (0.1 U/ml at 37°C for 3 h, Sigma Chemical, St Louis, MO) prior to electrophoresis on NuPAGE 3–8 % gradient gel and transferred to a nitrocellulose membrane. The membrane was then blocked in 2% nonfat dry milk in PBS containing 0.05% Tween-20 (PBST) for 1 h at room temperature with gentle agitation. After blocking, the blots were probed overnight with anti-CSPG-4 (1:10,000; ICN, Costa mesa, CA), anti-fibronectin (1:10,000; DAKO, Carpinteria, CA), or anti-tenascin (1:5000). Antibody was diluted in 1 % BSA in PBST overnight at 4°C with gentle agitation. After extensive washes in PBST, blots were incubated with HRP labeled secondary antibodies in 1% BSA in PBST for 1 h with agitation. Goat anti-rabbit-HRP (1:10,000) was used for Tenascin antibodies and Goat anti-Mouse (IgG+IgM) (1:10,000) was used for fibronectin and CSPG-4 and -6. The blots were again rinsed extensively in PBST and bands were visualized using the Renaissance chemiluminescence reagent from New England Nuclear (Boston, MA). Optical density measurements were made using a UVP Chemi-Imaging system.

For Immunoblotting for glutamine synthetase (GS), glutamate aspartate transporter (GLAST), glutamate transporter-1 (GLT-1), S-100B and protease-activated receptor (PAR-1), 10 μg of protein were analyzed. Blots were incubated in rabbit anti-GLT-1 (1:1000), rabbit anti-GLAST (1:1000) (Alpha Diagnostic International, San Antonio, TX), mouse anti-GS (Chemicon International, 1:2000), rabbit anti-PAR1 (Santa Cruz Biotechnology,1:1000), or mouse S-100B (1:1000) (Sigma chemical, St Louis, MO) antibody. Blots were stripped (30 min at 50°C in 62.5 mM Tris-HCl pH 6.8, 2% SDS, 100 mM 2-mercaptoethanol) and re-probed with anti-β-tubulin antibody (1:1000, Santa Cruz Biotechnology, Santa Cruz, CA) to confirm equal loading of proteins.

## Results

### Absence of IL-1R1 signaling leads to attenuated hypertrophy of astrocytes and delayed induction of GFAP (Fig. 1 and 2)

**Figure 1:**
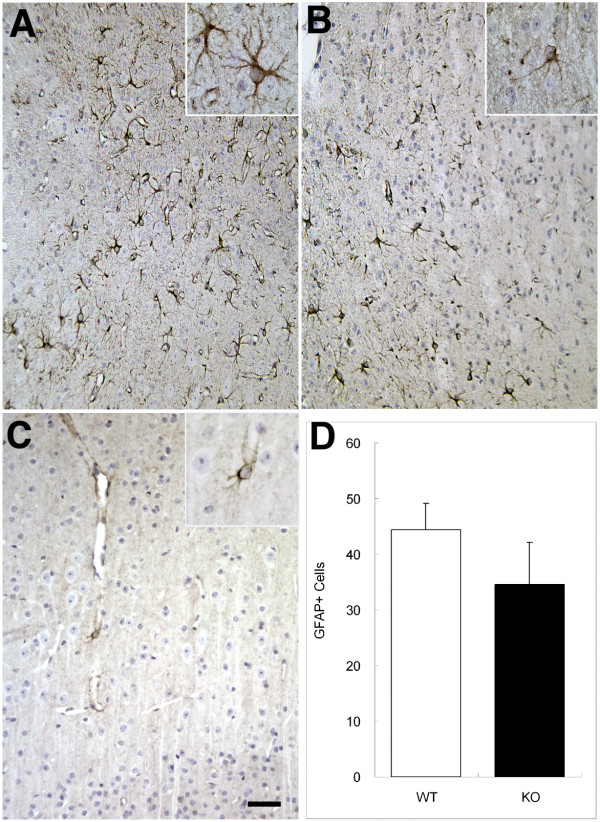
**Deletion of IL-1R1 reduces GFAP immunoreactivity but does not alter the number of GFAP+ astrocytes after a penetrating neocortical injury. **Adult wild-type mice (A and C) or age matched IL-1R1-null mice (B) received a penetrating brain injury to the somatosensory cortex. After 3 d, animals were sacrificed and processed for GFAP immunohistochemistry. Panels A and B were captured from layers 3–5 of the neocortex within the penumbra of the lesion whereas panel C depicts the contralateral hemisphere from the wt animal at 10×. Insets depict representative cells from WT or IL-1R1-null mice at 40×. Scale bar represents 50 μm. Counts of GFAP+ cells (D) were performed on photomicrographs taken in areas 240 μm away from the lesion site of brain sections from WT (n = 4) and IL-1R1-null (n = 3) animals at day 3 at 40×. The number of GFAP+ astrocytes from each picture was counted by an investigator blinded to their identity. Values represent the means ± S.E.M.

GFAP immunohistochemistry revealed that GFAP expression was attenuated in the IL-1RI-null mice compared to their WT counterparts following a neocortical stab wound (Fig 1). At 3 days post lesion, GFAP immunoreactivity was increased in both WT and null mice, but the response was markedly abrogated in IL-1R1-null mice. Astrocytes adjacent to the injury in the WT mice appeared hypertrophied and exhibited a dramatic increase in GFAP immunoreactivity (Fig 1A inset). In contrast, IL-1R1-null mice stained less robustly for GFAP and the astrocytes appeared on average smaller in size (Fig 1B inset). In the unlesioned cortice, GFAP+ cells are less frequently observed and appeared in similar size as seen in IL-1R1-null animals (Fig 1C inset). Quantifying the numbers of GFAP+ cells (Fig 1D) in the lesion penumbra revealed a trend towards the IL-1R1-null animals having fewer GFAP+ cells than the WT animals, but this trend was not statistically significant.

**Figure 2:**
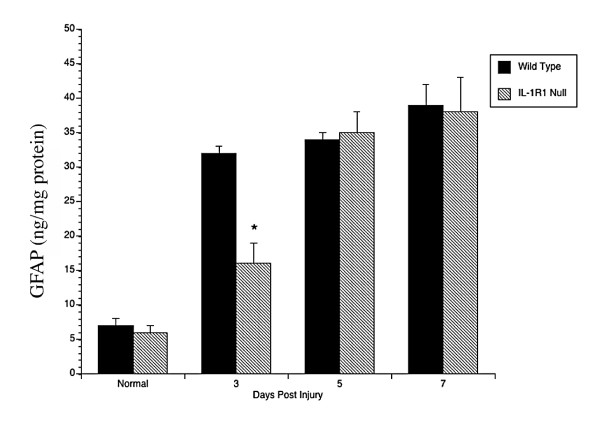
**The increase in GFAP protein is delayed in IL-1R1-null mutant mice vs WT mice after a penetrating brain injury. **GFAP levels were measured from lesioned neocortices by two-site ELISA at 3, 5 and 7 d after injury in wild-type or IL-1R1-null mice. Values represent the means ± S.E.M. from at least 6 mice per time point. p < 0.05 by Student t test.

An analysis of GFAP protein levels by using a two-site ELISA confirmed the immunohistochemical findings (Fig 2). At 3, 5 and 7 days after stab wound, GFAP expression was increased by stab wound injury in both WT and receptor-null mice. However, compared to the WT counterparts GFAP levels were attenuated at the early time point (3 days post lesion) in the receptor-null mice, but by 5 days of recovery GFAP achieved comparable levels to injured WT mice. Routine histological analyses did not reveal any obvious differences in the extent of the initial injuries sustained by the animals. Thus, these data show that the cellular expression of GFAP is delayed in the IL-1R1 null mice, but that a compensatory mechanism, such as the delayed production of other cytokines, eventually stimulates GFAP expression to the same level as induced in the wild-type animals [45].

### Induction of protease-activated receptor-1 (PAR-1) by stab wound injury is ablated by the deletion of IL-1R1 (Fig. 3 and 4)

**Figure 3:**
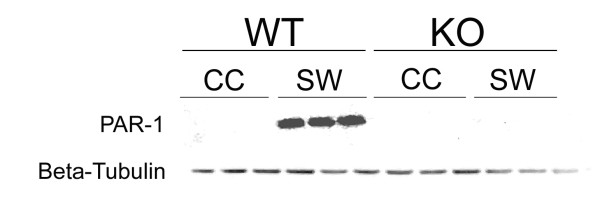
**Thrombin receptor 1 (PAR-1) protein is depressed in IL-1R1-null mice after a stab wound injury. **Tissues from 3 wild-type (WT) and 3 IL-1R1null mice (KO) at 3 d after stab wound (SW) were analyzed by Western blot for PAR-1. The blot was reprobed for β-tubulin to confirm equal protein loading.

**Figure 4:**
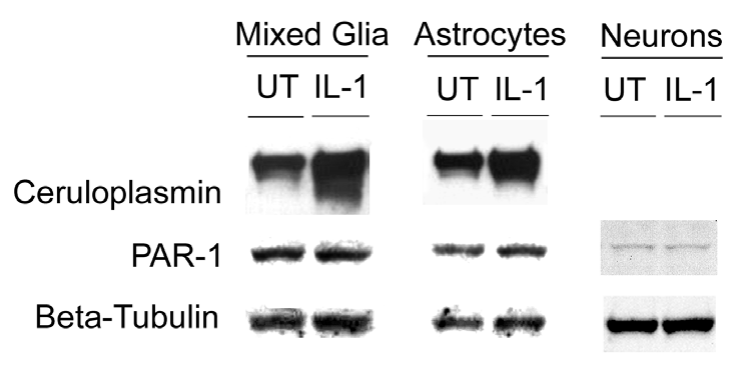
**IL-1β slightly increases PAR-1 protein expression in the primary astrocyte cultures, but not that in neuronal nor mixed glial cultures. **Mouse cortical neuronal, mixed glial and astrocyte cultures were treated with 5 ng/ml of rmIL-1β for 24 hr and 10 μg of protein was analyzed by Western blot. Increased ceruloplasmin expression demonstrated that the mixed glia and astrocytes responded to IL-1β. The blot was reprobed for β-tubulin to confirm equal protein loading. Data are representative of results obtained from three independent experiments.

During injury thrombin is released and cleaves the protease-activated receptors (PARs), which subsequently induce plasma extravasation and inflammation. Activated thrombin receptors also stimulate glial cell proliferation [46]. Therefore, we analyzed the expression of PAR-1 following penetrating brain injury. The PAR-1 expression was dramatically increased in the WT mice at 3 days post injury (Fig. 3). By contrast, PAR-1 protein was not induced and remained undetectable in the IL-1R1-null mice. To elucidate which cell type expresses PAR-1 protein, we performed immunofluorescence staining of PAR-1 on the brain sections. However, the immunofluorescence lacked the sensitivity and specificity to determine which cell type expresses PAR-1 after neocortical injury. Therefore, we performed *in vitro *studies to examine which brain cell increases PAR-1 expression in response to IL-1β stimulation. IL-1β at 5 ng/ml was used to stimulate primary cultures of mixed glia, astrocytes, microglia and cortical neurons, and the expression of PAR-1 proteins was assayed. Upon stimulation with IL-1β, the expression of PAR-1 slightly increased in the astrocyte cultures, but not in mixed glial or cortical neuronal cultures (Fig. 4), and it was undetectable in the microglial culture (data not shown). To ensure that the astrocyte and mixed glial cultures were responding to IL-1β, the expression of ceruloplasmin (CP) was analyzed. As expected, IL-1β increased CP significantly in the astrocyte and mixed glial cultures.

### Extracellular matrix molecules are independent of IL-1R1 (Fig. 5 and 6)

**Figure 5:**
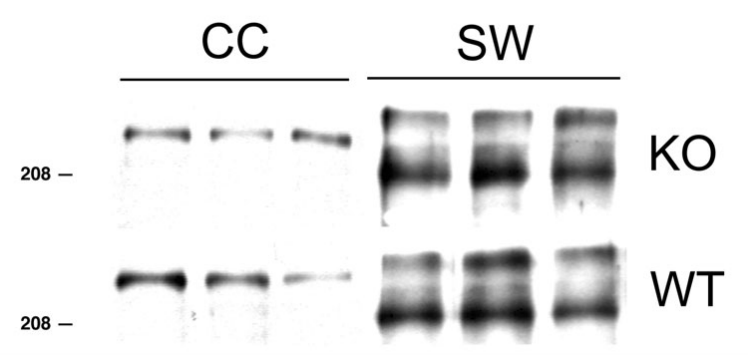
**Extracellular matrix protein, tenascin-c, is induced by a stab wound injury. **Neocortices from 3 wild-type (WT) and 3 IL-1R1-null mice (KO) at 5 d after stab wound or protein samples from the contralateral cortex were analyzed by Western blot for tenascin-C.

Extracellular matrix (ECM) molecules play an important role in mediating the wound-healing process in the body, and are essential components of glial scars. In adult CNS, ECM molecules, such as chondroitin sulfate proteoglycans (CSPG) and tenascin, are expressed at low levels; however, injury can elicit a prominent increase in their expression, which is primarily associated with reactive astrocytes surrounding the injury site. Thus, we analyzed the protein levels of tenascin-c and CSPG-4 family. Tenascin-c resolved as a single band by Western blot in the unlesioned brain at approximately 220 kDa (Fig. 5). Following the stab wound injury, tenascin resolved as two bands at approximately 208 and 240 kDa. However, there was no difference in the induced level of tenasin-c between the WT and the IL-1R1-null mice.

**Figure 6:**
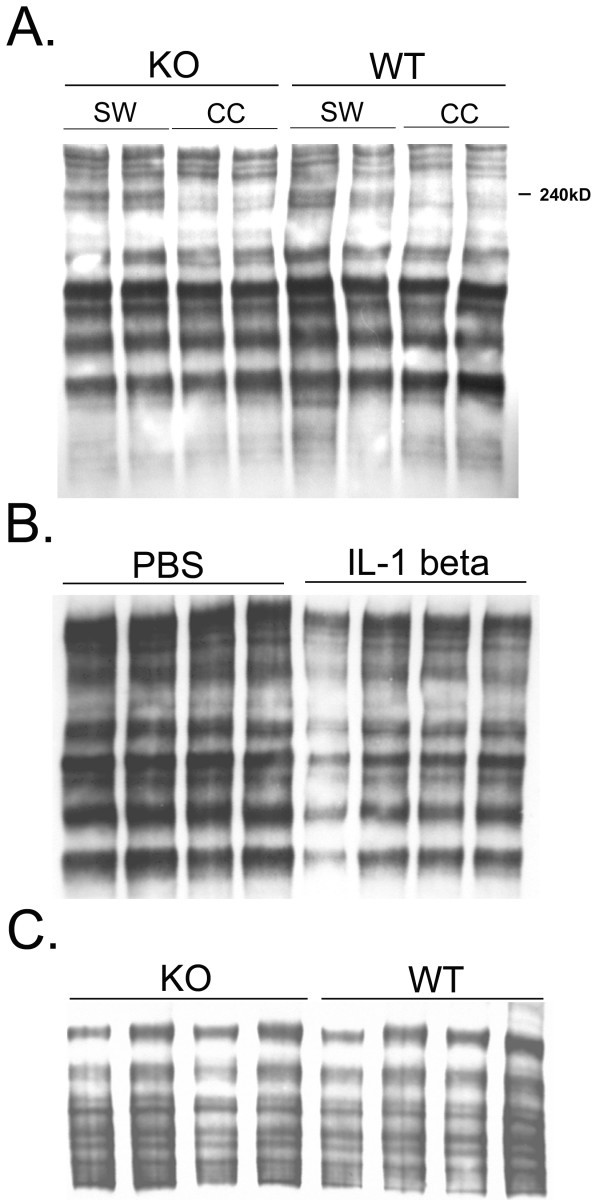
**Chondroitin sulfate proteoglycans-4 (CSPG-4) is induced by stab wounds, but not by IL-1β. A**, Neocortices from 2 wild-type (WT) and 2 IL-1R1-null mice (KO) at 10 d after stab wound or protein samples from the contralateral cortex were analyzed by Western blot for CSPG-4. Each lane represents protein from an individual animal. **B**, Samples from injected neocortices were homogenized in chondroitinase ABC and analyzed by Western Blot for CSPG-4. Each lane represents an individual WT animal that received either IL-1β or PBS. **C**, IL-1β was injected into WT or IL-1R1-null mice. Neocortices from 4 WT and 4 IL-1R1-null mice at 5 d after injecting 1 ng IL-1β were analyzed by Western blot for CSPG-4. Each lane represents protein from an individual animal.

Similarly, the expression of a CSPG-4 protein of approximate molecular weight of 240 kDa was increased after the injury, but there was no difference in the expression between WT and IL-1R1-null animals (Fig. 6A). To confirm that the induction of CSPG-4 was independent of IL-1β, we injected IL-1β into the neocortex of WT and IL-1R1-null mice and analyzed CSPG-4 levels after 5 days. Consistently, IL-1β did not induce CSPG-4 expression in WT or IL-1R1-null mice (Fig. 6B and 6C).

### Several astrocytic functions are also independent of IL-1R1 (Fig. 7)

**Figure 7:**
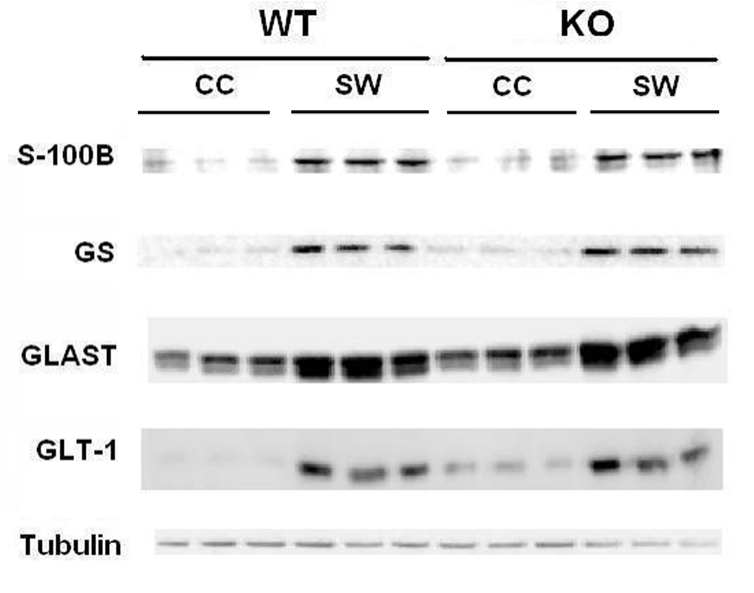
**Glutamate transporters, GLAST and GLT-1, glutamine synthetase, GS, and S-100B are upregulated in both WT and IL -1R1-null mice after a penetrating brain injury. **GLAST, GLT-1, GS and S-100B protein expression was analyzed by Western Blot on tissues from the lesioned cortices of wild-type mice (WT-SW), an equivalent region of unlesioned contralateral cortices of the same wild-type animal (WT-CC), the lesioned cortices of receptor-null mice (KO-SW), and an equivalent region of unlesioned contralateral cortices of the same receptor-null animal (KO-CC). Blots were re-probed for β-tubulin to establish equal protein loading on the gel. Lanes represent samples from 3 individual WT animals at 3 d after stab wound.

To assess the functional state of astrocytes after traumatic brain injury, we analyzed the expression of two glutamate transporters, glutamate aspartate transporter (GLAST) and glutamate transporter-1 (GLT-1/EAAT2), the glutamate transaminase, glutamine synthetase (GS) and the calcium regulatory protein S-100B. These proteins enable astrocytes to regulate the levels of two important signaling molecules in the brain, glutamate and calcium. Our results show that in both WT and receptor-null mice, stab wound injury increased GLAST, GLT-1, GS and S-100B protein expression at 3 day post injury by 8, 6, 4 and 12 fold, respectively (Fig. 7). However, neither the basal nor induced levels of these proteins were different between the WT and the receptor-null mice. Although we observed decreased GFAP expression at this time point, our data indicate that there is reduced GFAP per cell rather than fewer astrocytes. Thus, these results suggest that several astrocytic physiological functions, such as the capacity to clear glutamate, synthesize glutamine from glutamate and buffer levels of calcium, do not depend upon IL-1 signaling through IL-1R1 in either the normal or injured state.

## Discussion

IL-1β coordinates many of the initial and late stages of cellular responses to injury. Since IL-1β is usually present in elevated quantities in and around sites of injury, it has been cast in a negative light in the context of CNS injury and diseases [11,13,47-49]. In particular, since IL-1 can induce many pro-inflammatory mediators causing undesirable effects, it is regarded as an undesirable injury-associated cytokine [20,21,50,51]. Furthermore, IL-1R1 is essential for the activation of microglia and the induction of multiple pro-inflammatory mediators in response to brain injury [31-33]. Altogether, these studies suggest that the signaling of IL-1 through IL-1R1 can be deleterious through both direct and indirect actions.

Astrocytes play a major role in restoring homeostasis to the damaged brain and IL-1β regulates multiple astrocytic responses after injury [52]. The data presented in this communication demonstrate that several aspects of the astroglial response subsequent to CNS trauma require IL-1 signaling through the IL-1R1; however, quite a few adaptive physiological functions of astrocytes are independent of IL-1R1 signaling. In summary, this study on the effect of a penetrating brain injury in mice lacking IL-1R1 demonstrates that IL-1R1 deletion results in: 1) attenuated hypertrophy of astrocytes; 2) delayed cellular GFAP induction; 3) diminished induction of PAR1; 4) intact induction of extracellular matrix proteins and 5) intact induction of glutamate transporters, glutamine synthetase and S-100B.

The induced levels of the protease-activated receptor, PAR-1, were significantly attenuated in IL-1R1-null mice. Thrombin, a serine protease generated by cleaving pro-thrombin, is an essential component of the coagulation cascade. It is produced in the brain either immediately after a cerebral hemorrhage (primary or secondary to brain trauma) or after the blood-brain barrier (BBB) breakdown that occurs following brain injury [53]. Evidence, both *in vivo *[46,54,55] and *in vitro *[56,57] indicate that high levels of thrombin within brain parenchyma can be deleterious. A recent report documents upregulated PAR-1 expression in astrocytes during HIV encephalitis [58]. Our findings suggest that blocking IL-1 signaling via IL-1R1 may attenuate the activation of PAR-1 after brain injury. To determine which cell type is induced to express PAR-1, the effects of IL-1β on PAR-1 expression were assessed *in vitro*. The level of PAR-1 protein expression after IL-1β stimulation was examined in the mixed glial, enriched astrocyte, enriched microglial and cortical neuronal cultures. The level of PAR-1 expression trended towards increasing in the astrocyte cultures; the level was unchanged in mixed glial cultures, the level was very low in the cortical neuronal cultures and below the level of detection in the microglial cultures. Altogether, these results suggest that brain cells are not responsible for the induction of PAR-1 expression after traumatic brain injury. Other cell types, such as endothelial cells or infiltrating monocytes are likely candidates [59,60]. As the brains were not perfused prior to extracting tissue for analysis, therefore, the observed PAR-1 could have been in the vascular compartment.

Extracellular matrix (ECM) molecules, including CSPGs and tenascin, are important participants in the wound-healing process. They are expressed at low levels in the normal brain and are induced by injury. In a damaged brain, this increase is primarily associated with reactive glia that surround the injury site [61]. The astrocytes respond to CNS injury by forming "astroglial scars", which can become a barrier to regenerating axons. It has been observed that axons fail to regenerate past a lesion site, even in the absence of a recognizable glial scar [62]. This suggests that reactive glia establish a biochemical rather than a physical barrier that inhibits axonal regeneration. Following CNS injury, CSPGs are upregulated in areas of reactive gliosis and multiple molecular species are induced [61,63,64]. These injury-induced CSPGs inhibit neurite outgrowth both by directly acting on receptors present on growth cones as well as by indirectly altering the actions of growth-promoting factors [65,66]. Furthermore, the CNS-specific CSPG core proteins brevican and phosphacan are primarily expressed by astrocytes [67-69], whereas the neuroglycan 2 (NG2) CSPG is produced by a unique population of glial cells termed polydendrocytes [70,71]. NG2 mRNA and protein levels are induced after many types of CNS injury [72]. Neurocan is another CSPG distributed throughout the developing CNS [69]. Although neurocan is initially localized to neurons [73], it is also expressed by astrocytes [74].

In the present study we confirmed that CSPGs are induced by traumatic brain injury, and also found that the injury-induced expression of CSPGs is unaffected by IL-1R1 deletion. One logical mechanism is that IL-1 signals through an alternative receptor than IL-1R1, and hence deleting the IL-1R1 does not affect signaling through that receptor. Or, the induction of CSPGs is mediated by other factors. However, to date we have no direct evidence from our studies for an alternative IL-1 receptor mediating the effect of IL-1. Furthermore, if an alternative receptor acts to induce the expression of CSPGs, we should have seen an increased expression of the CSPGs when we directly injected the IL-1 into the IL-1R1-null mice. The absence of such a response suggests that other factors are responsible to the induction of CSPGs in response to injury. A strong candidate is transforming growth factor-beta (TGF-β) [75].

Neuronal dysfunction subsequent to brain damage causes the release of glutamate, which can lead to secondary excitotoxic neuronal death and death of oligodendroglial progenitors. Astrocytes regulate glutamate levels by actively removing it from the extracellular space and converting it to glutamine. The capacity of astrocytes to reduce extracellular levels of glutamate dramatically impacts the extent of neuronal and oligodendroglial damage after an insult. Astrocytes possess two glutamate transporters that sequester excess glutamate, GLT-1 and GLAST, and glutamine synthetase, which converts glutamate to glutamine. Here we demonstrate that a penetrating brain injury increases the expression of GLT-1 and GLAST. Previous studies also have shown increases in these transporters as a result of other injury paradigms. For instance, GLT-1 levels increase 2.5 fold above the control three days after the trauma caused by transplanting E18 neocortical tissue into rat cortex [76]. Similarly, GLT-1 and GLAST mRNA expression are induced after cultured astrocytes are physically traumatized [77,78]. Studies from our lab indicate that there is a dramatic induction of GLAST protein in WT and IL-1R1-null mice after a mild hypoxic/ischemic insult (Sen et al. unpublished observation). In addition, the calcium regulatory protein S-100B was upregulated by the stab wound injury, but the levels of expression were not different between WT and IL-1R1-null mice. S-100B can affect a number of calcium regulated enzymes within astrocytes and it also can be secreted from astrocytes to serve as an intercellular signal between glial cells and neurons [38,40]. Thus, a neocortical stab wound injury induces the expression of GLT-1, GLAST, the GS, and S-100B, but our data indicate that this induction is independent of IL-1R1.

Data presented in this communication and from previous studies in our laboratory support the concept that blocking IL-1 signaling through IL-1RI will reduce damage caused by injury or disease. Our previous studies have shown that the induction of NGF and ceruloplasmin is preserved when this receptor is deleted [31,34]. In this paper we demonstrate that IL-1R1 deletion has minimal effects on glutamate homeostatic proteins and calcium binding proteins in astrocytes. As numerous studies have provided rationale for antagonizing the IL-1R1 to prevent damage to CNS neurons and glia, a concern has been that the adaptive responses of the astrocytes that occur subsequent to IL-1 stimulation will be lost. In the present study we show that abrogating IL-1R1 signaling will not have any direct effect on sequestering and detoxifying glutamate nor on S-100B-mediated signaling in the brain as these functions are preserved when this receptor is blocked.

## Conclusion

We show that a number of astrocytic functions, including the increased capacity to buffer glutamate and the increased capacity for S-100B signaling are preserved when the IL-1RI is genetically ablated. On the other hand, the absence of IL-1R1 signaling results in attenuated hypertrophy of astrocytes, delayed induction of cellular GFAP, decreased induction of PAR-1 and unperturbed production of extracellular matrix proteins. In a previous study, we showed that abrogated IL-1R1 signaling decreases the responsiveness of microglia and macrophages to injury and lowers the basal and induced levels of cyclooxygenase-2, IL-1 and IL-6 [79]; these results suggest that antagonizing IL-1R1 decreases inflammatory responses after injury. Altogether, these data provide important support for the development of therapies designed to antagonize this receptor. Our research suggests that these strategies may reduce inflammation and preserve the adaptive gain of physiological functions by astrocytes in the central nervous system.

## Competing interests

The author(s) declare that they have no competing interests.

## Authors' contributions

HL participated in the design of the study, conducted the experiments on the primary cultures, performed the statistical analysis and prepared the manuscript. AB carried out the stab wound surgeries and performed Western blot analyses and immunohistochemistry on tissue samples after injury. CD performed ECM Western analysis and MC conducted Western analysis of tenascin and analysis of GFAP by Western and ELISA. JKK and SWL designed and supervised the studies. All authors have read and approved of the final manuscript.
